# Hand Hygiene Accuracy of Students and Staff at a Dutch Dental School

**DOI:** 10.1111/eje.70097

**Published:** 2026-01-21

**Authors:** Ilona Francisca Persoon, Catherine Minke Charlotte Volgenant

**Affiliations:** ^1^ Department of Preventive Dentistry, Academic Centre for Dentistry Amsterdam (ACTA) University of Amsterdam and Vrije Universiteit Amsterdam Amsterdam the Netherlands; ^2^ Department of Cariology, Academic Centre for Dentistry Amsterdam (ACTA) University of Amsterdam and Vrije Universiteit Amsterdam Amsterdam the Netherlands

**Keywords:** dental school, dental students, dentistry, hand hygiene, infection control

## Abstract

**Introduction:**

Oral health care professionals continuously come into contact with bodily fluids containing many pathogens. Hand hygiene should be used to prevent cross transmission. We aimed to examine the accuracy of application of alcohol‐based hand rub (ABHR) according to the WHO hand hygiene steps at a dental school.

**Materials and Methods:**

Students and staff filled out a short questionnaire to obtain demographic data and answered questions on their perceived efficacy and knowledge of their hand hygiene. Participants rubbed their hands as they usually do when working in the clinic using an ABHR supplemented with a fluorescent marker, after which the dorsal and palmar sides of both hands were photographed. Photographs were scored according to the steps of the WHO guideline. Parts of the hands were scored as completely or insufficiently covered with ABHR.

**Results:**

The average hand hygiene accuracy of Dutch dental students and staff was 59%. Only 2.8% of them had excellent accuracy of hand hygiene. The most inefficiently performed steps were rubbing the thumb and rubbing the backs of the fingers to opposing palms. No differences could be observed according to student training year or being student or staff.

**Conclusion:**

Overall, hand hygiene accuracy at a Dutch dental school was comparable to that observed in other health care sectors. Only a few participants demonstrated excellent technique and performance was not associated with educational level. These findings highlight the need for greater emphasis on hand hygiene in dental curricula as well as complementary strategies for improving hand hygiene.

## 1 Introduction

Hand hygiene is a cost‐effective and universally available measure to prevent dispersion of microorganisms and health care‐associated infections, which has received extra attention during the COVID‐19 pandemic [1]. Oral health care workers frequently come into contact with sources of pathogenic microorganisms, such as mucosa, saliva and blood. These reservoirs might contain human immunodeficiency virus, hepatitis B virus, respiratory viruses or (methicillin resistant) *Staphylococcus aureus* [2]. Vice versa, microorganisms from the oral health care worker's skin could be transferred to the patient's skin or oral cavity. To prevent cross transmission, oral health care workers wear nonsterile examination gloves when in contact with patients and frequently apply hand hygiene [3]. Both compliance and technique contribute to effective hand hygiene. Most often, hand disinfection using alcohol‐based hand rub (ABHR) is applied. Its main ingredient is alcohol, which is highly efficacious against the most prevalent pathogenic microorganisms [4]. The World Health Organization (WHO) recommends when and how to disinfect the complete hand surface using five steps [3]. The WHO also makes recommendations on how to teach and promote hand hygiene [3].

Less than 10% of health care workers are able to obtain full coverage with ABHR, maintaining the risk of transmitting pathogenic microorganisms [5, 6]. Studies at dental schools have found hand hygiene accuracy rates of 37%–78% using direct observation [7, 8, 9]. However, this method is time‐consuming and subject to error [1]. To our knowledge, no studies examining the hand hygiene technique in detail in dentistry and dental schools have been published so far. Therefore, we aimed to examine how accurately students and staff performed the WHO hand hygiene steps at a dental school using fluorescent ABHR. Moreover, the effect of training on this accuracy was assessed.

## 2 Materials and Methods

The study was conducted at the Academic Centre for Dentistry Amsterdam (ACTA; The Netherlands). This dental school trains approximately 900 students and has 450 employees. First‐year students receive theoretical training on hand hygiene according to the WHO guideline [3]. Hand hygiene instructions are available next to each hand washing station at the clinic. Relevant protocols are also available on the closed electronic network. All clinical students and staff are tested regarding these protocols as part of a yearly digital infection control exam.

The study protocol was approved by the ACTA ethical committee (2018020) and was exempted from the Medical Research Involving Human Subjects Act. All participants provided written informed consent.

Dental students and clinical staff were randomly approached at the clinic and asked to participate anonymously. The group size was based on a convenient sample of available students, evenly distributed per training year. Participants filled out a short questionnaire to obtain demographic data and to assess their knowledge on hand hygiene, using 12 agree/disagree depositions on hand hygiene facts and reported compliance. Hands were rubbed as participants usually do when working in the clinic using 3 mL of ABHR supplemented with a fluorescent marker (Schülke optics; Schülke & Mayr GmbH, Germany). In a dark environment, hands were exposed to blacklight (Eurolite UV spot blacklight 230 V/25 W, Eurolite, Canada). Areas covered by ABHR turned white, whereas unrubbed areas remained dark [6]. In a standardised setting, photographs were taken from the dorsal and palmar sides of both hands (Canon EOS 700D + EF‐S 18–55 mm, Canon, Japan).

Photographs were independently scored by two examiners (I.F.P. and C.M.C.V.) according to the five steps of the WHO guideline and the wrists for the right and left hand [3]. In order to identify these steps, the dorsum of each hand was scored for three separate parts (dorsum of main body of the hand including wrist, and dorsum of finger and thumb) and the palm of each hand was scored for four separate parts (palmar side of the main body of the hand including wrist, finger, top of the finger and thumb). These scores were computed into results for the six steps of the WHO guideline. Parts of the hand were scored as completely or insufficiently covered with ABHR. When the examiners disagreed, that step was scored as insufficient. Hand hygiene accuracy was determined by ABHR coverage, which was defined as the number of the 12 assessed steps of both hands fully covered with ABHR. Participants that had completely covered all 12 steps were considered to have an excellent hand hygiene technique. Intra‐examiner reliability was assessed by scoring the photographs of 30 participants in duplicate [10]. Inter‐examiner reliability was assessed across the whole dataset.

Statistical analyses were performed using SPSS Statistics 27.0 (International Business Machines Corporation, USA). Cohen's kappa was used to determine intra‐examiner and inter‐examiner reliability. The Mann–Whitney *U*‐test was used to compare ABHR coverage of students to staff and left to right hands. This test was also used to determine the effect of sex, dominant hand and questionnaire items on ABHR coverage. Students of different training years were compared using the Kruskall–Wallis test. The effect of ABHR coverage on knowledge and on progression along the 6 years of students' dental study was assessed using the Spearman rank correlation. The level of significance was set at *α* = 0.05.

## 3 Results

In total, 221 dental students (77% female, 89% right‐handed) participated, representing each of the 6 training years (respectively 44, 33, 58, 26, 34 and 26 participants). The 29 participating staff members (65% female, 83% right‐handed) were working as dental assistants, oral hygienists or dentists. Most participants (both staff and students) wore size S gloves (4% XS; 55% S; 31% M; 9% L; 1% XL).

The intra‐examiner and inter‐examiner reliability were good (IFP Cohen's *κ* = 0.70; CMCV Cohen's *κ* = 0.79; inter‐examiner Cohen's *κ* = 0.70). Participants had complete ABHR coverage in 7.1 ± 2.0 out of 12 steps. Only 2.8% of participants had an excellent hand hygiene technique with full ABHR coverage (Figure 1). The most inaccurately performed steps were rubbing the thumb and backside of the fingers to opposing palms (Table 1). ABHR coverage was independent of sex, dominant hand, left or right hand and glove size (resp. *p* = 0.40, *p* = 0.59, *p* = 0.68 and *p* = 0.60). Students had similar ABHR coverage to staff (7.0 ± 1.9 vs. 7.7 ± 2.2, *p* = 0.14). Also, students of none of the training years performed significantly better than others (*p* = 0.67), and similarly, students did not score better if they were further along their training (*p* = 0.84).

**FIGURE 1:**
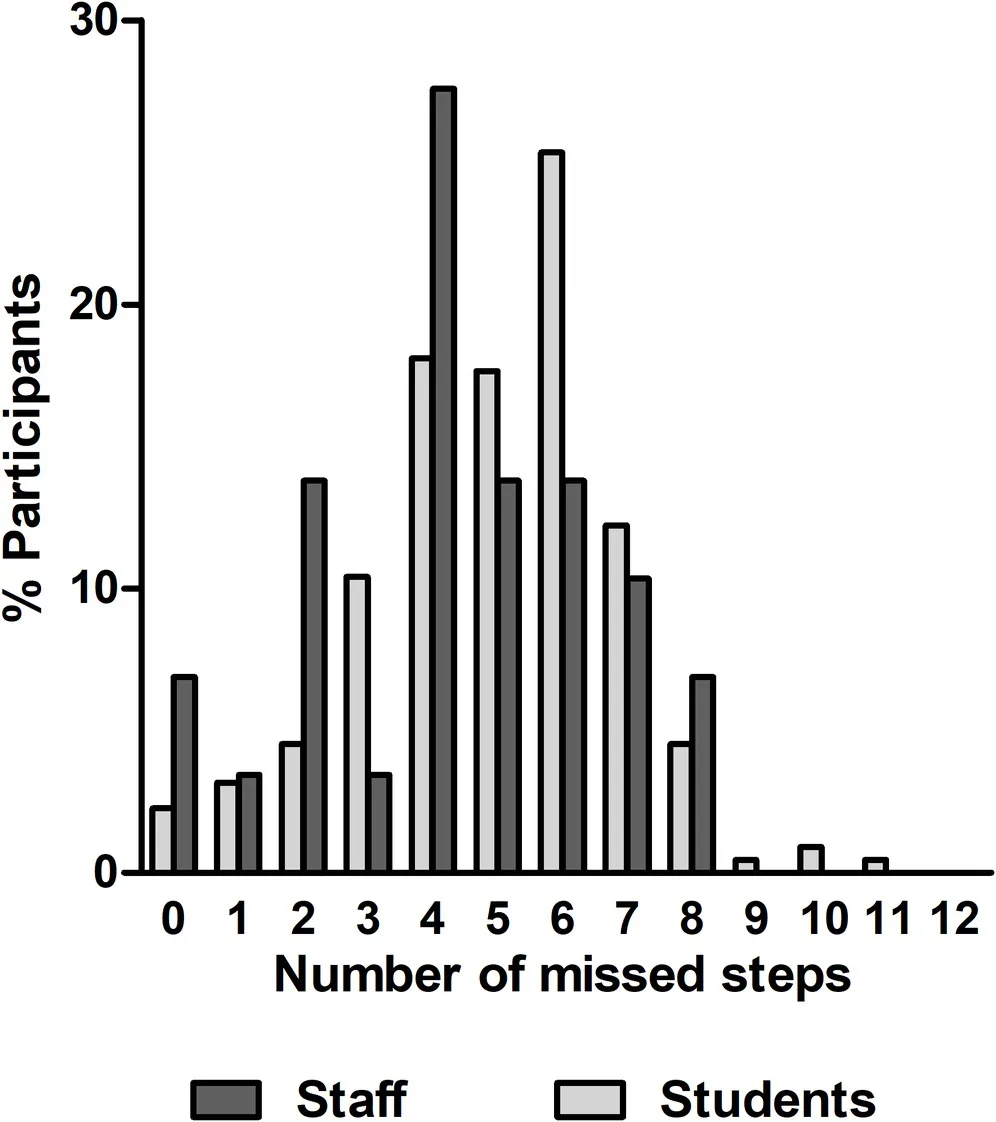
Number of missed steps of the 6‐step hand hygiene technique. Data of the left and right hand are displayed separately (total of 12 steps).

**TABLE 1:** Insufficient hand hygiene steps.

	Palm to palm	Palm over dorsum	Fingers interlaced	Fingers interlocked	Rubbing thumbs	Rubbing fingertips
Student (*N* = 221)	17%	56%	3%	81%	83%	9%
Staff (*N* = 29)	22%	38%	2%	72%	81%	0%

*Note:* Percentage of participants that had insufficiently covered this part of the hand with ABHR, that is, that did not perform this step of the 6‐step hand hygiene technique properly. Data are combined for the left and the right hand.

Despite their similar hand hygiene accuracy, students had lower hand hygiene knowledge scores than staff (55.1% ± 12.6% vs. 63.6% ± 15.6%, *p* = 0.008). Though overall, ABHR coverage was positively associated with hand hygiene knowledge (*r*_s_ = 0.20, *p* = 0.002). The 66% participants that claimed to know hand hygiene protocols and to adhere to these protocols had only borderline significantly better ABHR coverage than the other 33% participants (7.3 ± 2.0 vs. 6.9 ± 1.8, *p* = 0.05). Interestingly, 44% of the participants indicated that they would like more training on hand hygiene in the curriculum, but they already had significantly better ABHR coverage (7.8 ± 2.0 vs. 7.1 ± 2.0, *p* = 0.044).

## 4 Discussion

In this study, 2.8% of 250 dental students and staff achieved full coverage of the hands with ABHR. This result is comparable to studies on hospital staff using similar assessment methods, which detected no mistakes in hand hygiene in 7.9% and 9.5% of participants [5, 6]. Studies using observation at dental schools yielded a hand hygiene accuracy of 37%–78% in a clinical setting [7, 8, 9]. The average accuracy rate in the current study was in a similar range with 59%. Furthermore, the precise assessment method enabled us to provide more detailed outcomes. Most participants insufficiently rubbed the thumbs and the dorsal side of the fingers or hands, which is in accordance with other studies [5, 6]. These steps require a specific technique in order to achieve full coverage with ABHR and may, therefore, require more diligence to perform [1]. Experimental research is looking into simplifying the 5‐step technique, as advocated by the WHO, to fewer steps, a different order of steps or shorter duration [1].

In this study, participants were offered a standardised quantity of ABHR as dictated by international norms (EN 1500), which was more than sufficient to ensure full coverage of both hands. Although this was not part of the questionnaire, several participants informally noted that the amount of ABHR supplied in the research was considerably more than they typically use in daily clinical practice. Additionally, hand size did not affect surface coverage, which may indicate abundant supply of ABHR. Most health care workers usually apply a single dose of ABHR, regardless of the volume released by the dispenser [11]. The same study stated that 3 mL as recommended by international norms is superabundant for most hands. However, they still observed that many areas of the hands were not covered with ABHR. It has been shown that if a large quantity of ABHR is automatically supplied from dispensers, this promotes a longer application and enables enlarged surface coverage [12]. Furthermore, participants were only assessed once without the time‐constraint or focus elsewhere as experienced during patient treatment. Combined with the presence of the observers, this might have enforced extra diligence with the participants in performing hand hygiene. This Hawthorne effect has been observed in studies into hand hygiene compliance [13, 14]. Therefore, the results may present an overestimation of accuracy of hand hygiene accuracy and external validity in daily clinical practice might be limited. However, the methodology was standardised and reproducible, and the results remain valid within the context of the study design.

In the current study, fluorescence techniques with a marker were used to assess hand hygiene accuracy. This method is widely used due to its simplicity and visual attractiveness. However, the inter‐examiner reliability can vary a lot with this method, although automated assessment can improve the reliability [15, 16]. Combining the fluorescent techniques with another method such as microbiological validation could enhance the reliability of the hand hygiene assessments. The advantage of incorporating microbiological validation in the assessment of hand hygiene assessments is that it not only evaluates the technique of hand hygiene but also includes whether the ultimate effect of achieving clean hands is accomplished. More advanced technologies are being developed to assess hand hygiene accuracy, such as by video analysis or sensor armbands [17, 18]. Thus far, fluorescent techniques provide a high‐throughput and accessible alternative.

Although we did not find statistically significant differences in hand hygiene accuracy based on sex or dominant hand, these variables may still be relevant in understanding individual technique. Previous research has shown that female students tend to perform better than male students in terms of rubbing quality and surface coverage [19, 20]. Educators may consider these findings when designing hand hygiene training. In our study, no differences were observed between the dominant and non‐dominant hand, which is consistent with findings from other studies using fluorescent ABHR [20]. This suggests that hand dominance may not play a major role in coverage accuracy, although further research is needed to confirm this in larger and more diverse populations.

The effect of clinical experience and knowledge on hand hygiene accuracy remains ambiguous. Dental students have been found to remove fewer bacteria by surgical handwashing with increasing clinical experience, although no effect of clinical experience could be found in the current and other studies [8, 21, 22]. However, staff have also been shown to comply more frequently with guidelines [7]. Furthermore, the current study and Thivichon‐Prince et al. found that more knowledge on hand hygiene was associated with increased hand hygiene accuracy [7]. Other studies were unable to corroborate this [8, 21]. One of the pillars of the WHO multimodal strategy for enhancing hand hygiene is education. It seems that hand hygiene is not a skill that automatically increases with clinical experience but requires that training should be specifically aimed at hand hygiene and should be repeated often [13, 23]. How education can contribute to performing hand hygiene still remains a subject for further research [1]. Although education can contribute to hand hygiene accuracy, no specific teaching modalities can be recommended [24]. Furthermore, other strategies are proven to be part of a successful implementation plan, including system change, providing evaluation and feedback, reminders in the workplace and installing a safety climate [25].

While this study offers valuable insights into hand hygiene accuracy at a dental school, certain limitations should be acknowledged. It was conducted at a single centre with a limited sample size, which may affect the generalizability of the findings, since a dental school is a very specific environment focusing on education compared to a general dental clinic or a general hospital. Additionally, participants were aware that their hand hygiene performance was being assessed, potentially leading to the Hawthorne effect. We consider this study a pilot for future research involving larger and more diverse populations, multiple institutions and varied clinical settings (e.g., educational clinics versus professional dental practices). Further studies may also compare hand hygiene accuracy in dental care with general health care settings to better understand discipline‐specific challenges and opportunities.

Determining whether the barriers experienced by dental health care workers are similar to those experienced by other health care workers could aid in identifying which barriers to address to improve hand hygiene compliance by dental health care workers. Identifying these barriers is also part of the strategy to tailor the educational program to a specific health care facility as advocated by the WHO [3]. Perhaps the recent pandemic has provided an extra incentive and training for applying hand hygiene. Proper hand hygiene is an easily accessible and available measure against cross contamination, health care associated infections and the spread of antimicrobial resistance [1].

## 5 Conclusion

The average hand hygiene accuracy was 59% at a Dutch dental school, with only 2.8% of the participants showing excellent technique. Students did not have better ABHR coverage if they were further along in their training and performed similarly to staff. These findings underscore the need for more attention to hand hygiene technique in dental education. As there is limited research on the accuracy of hand hygiene in dentistry, this study provides a valuable starting point for further investigation. Since oral health care professionals continuously come into contact with bodily fluids containing many pathogens, future research should focus on educational interventions and other strategies for improving hand hygiene. Successful implementation is key for dental schools with potential relevance for other institutions and private practices aiming to optimise infection control performance.

## Funding

The authors have nothing to report.

## Conflicts of Interest

The authors declare no conflicts of interest.

## Data Availability

The data that support the findings of this study are available from the corresponding author upon reasonable request.
